# Predictive biomarkers for immunotherapy response in endometrial cancer: current insights and future directions

**DOI:** 10.37349/etat.2026.1002381

**Published:** 2026-07-23

**Authors:** Giuliana Pavone, Alessandra Spata, Marialuisa Puglisi, Vincenza Ricco, Ilaria Colombo

**Affiliations:** N.N. Petrov Research Institute of Oncology, Russian Federation; ^1^Department of Clinical and Experimental Medicine, University of Catania, 95131 Catania, Italy; ^2^University Oncology Unit, Humanitas Istituto Clinico Catanese, 95045 Misterbianco, Italy; ^3^Department of Medical Oncology, Ospedale di Rho, ASST Rhodense, 20024 Milan, Italy; ^4^Medical Oncology Unit, Department of Human Pathology “G. Barresi”, University of Messina, 98122 Messina, Italy; ^5^Oncology Institute of Southern Switzerland (IOSI), EOC, 6500 Bellinzona, Switzerland

**Keywords:** endometrial cancer, immunotherapy, immune checkpoint inhibitors, microsatellite instability, biomarkers

## Abstract

In recent years, immunotherapy has modified the treatment landscape of advanced and recurrent endometrial cancer (a/rEC), particularly for patients with defective mismatch repair and microsatellite instability-high (dMMR/MSI-H), significantly improving their outcomes. Its success in later treatment lines has led to its investigation and adoption as a first-line therapy, alone or with chemotherapy. However, despite the high and long-lasting efficacy of immune checkpoint inhibitors (ICIs) in dMMR/MSI-H EC, not all patients benefit from this treatment, and reasons underscoring primary resistance in this setting remain poorly understood and are not yet incorporated into clinical decision-making. Additionally, the correlation between ICI response, tumor mutational burden (TMB), and PD-L1 expression, well-documented in other tumors, appears inconsistent in EC. While proficient mismatch repair and microsatellite stable (MMRp/MSS) EC remain an unmet medical need, some patients within this group still respond to ICIs. Although several biomarkers, including *TP53*, *BRCA*, and homologous recombination deficiency (HRD), have been investigated, none have proven to be definitively predictive. This review examines the relevant trials with ICIs as a single agent or in combination in EC and explores the available evidence on potential predictive biomarkers.

## Introduction

Endometrial cancer (EC) is the fourth most commonly diagnosed malignancy in women and represents the only gynaecologic cancer with a rising incidence [1]. According to the GLOBOCAN 2022 database, the global incidence of EC was estimated at 420,368 new cases, with 97,723 deaths attributed to the disease [2]. Despite its increasing prevalence, EC continues to exhibit relatively low mortality rates in Europe, ranging from 2.0 to 2.7 per 100,000 women. This is largely due to its often-indolent clinical course, early-stage detection in the majority of patients, and advances in molecular characterization, which have improved the development of more personalized therapeutic strategies [3].

Historically, EC was classified according to Bokhman’s dualistic model based on hormonal dependence and histopathological features [4]. However, this classification insufficiently captures the molecular heterogeneity of the disease. Subsequent genomic analyses from The Cancer Genome Atlas (TCGA) identified four molecular subgroups with distinct prognostic and biological characteristics, leading to the development of clinically applicable classifications such as the Proactive Molecular Risk Classifier for Endometrial Cancer (ProMisE) [5, 6]. Beyond prognostic implications, molecular classification has become increasingly relevant in predicting treatment response and guiding therapeutic strategies, particularly in the era of immunotherapy [7].

The defective mismatch repair (MMR) and microsatellite instability-high (dMMR/MSI-H) subgroup accounts for approximately 30% of EC and is characterized by defective DNA MMR mechanisms, elevated tumor mutational burden (TMB), and enhanced immune infiltration. These tumors frequently arise from *MLH1* promoter hypermethylation or, less commonly, germline or somatic mutations in MMR genes (*MLH1*, *MSH2*, *MSH6*, *PMS2*), resulting in biologically distinct immune microenvironments. Although generally associated with a favorable prognosis compared with p53-abnormal tumors, emerging evidence suggests heterogeneity in immune response and sensitivity to immune checkpoint inhibitors (ICIs) according to the underlying mechanism of MMR deficiency. This variability may partly explain differences in treatment outcomes observed within the dMMR/MSI-H population.

The *POLE*-ultramutated subtype accounts for around 10% of cases and is defined by somatic mutations in the exonuclease domain of the *POLE* gene, leading to an extremely high TMB but paradoxically low immune evasion. Despite high-grade histology, this group has an exceptionally favourable prognosis, likely due to strong immunogenicity and low metastatic potential [8]. This favorable prognosis has raised interest in treatment de-escalation strategies, while their high immunogenicity and elevated neoantigen load also suggest potential sensitivity to immunotherapy, although prospective evidence remains limited.

The p53-abnormal group comprises ~25% of ECs and is marked by mutations in the *TP53* gene and extensive copy number alterations, often correlating with a serous-like histology. These tumors are typically aggressive, with high-grade features, and are associated with a poor prognosis, reflecting their biologically aggressive behaviour [9].

The No Specific Molecular Profile (NSMP) category includes nearly 50% of cases. This heterogeneous group lacks defining molecular alterations such as *POLE* mutations, MMR deficiency, or *TP53* abnormalities. While often presenting as low-grade endometrioid tumors, NSMP cancers have an intermediate prognosis and remain the most molecularly diverse and clinically challenging to stratify. Recent evidence suggests that the expression status of estrogen receptor (ER) and progesterone receptor (PR) can further refine risk stratification within the NSMP group. In particular, ER- and PR-positive tumors tend to be associated with more favorable outcomes, whereas ER- and/or PR-negative NSMP cancers demonstrate more aggressive behavior and poorer prognosis [10].

These molecular distinctions have profoundly influenced therapeutic strategies, positioning predictive biomarkers as essential tools for identifying patients most likely to benefit from immunotherapy. However, despite the remarkable efficacy of ICIs in dMMR/MSI-H EC, substantial heterogeneity in treatment response persists, while predictive biomarkers beyond MMR status remain poorly defined. Understanding the biological and molecular determinants underlying immunotherapy sensitivity, therefore, represents a critical unmet need in EC.

In this review, a literature search was conducted using PubMed/MEDLINE, Embase, and Google Scholar to identify studies investigating predictive biomarkers and immunotherapy in EC. Articles published up to January 2026 were considered. Priority was given to phase II–III clinical trials, translational studies, post hoc biomarker analyses, international guidelines, and relevant congress presentations (ESMO, ASCO, SGO). Studies were selected based on their relevance to ICIs, predictive biomarkers, and treatment outcomes in advanced and recurrent EC (a/rEC). Additional studies were identified through manual review of reference lists. Given the narrative nature of this review, no formal systematic review methodology or quality assessment tool was applied.

## The role of immunotherapy in reshaping the treatment landscape of EC

The refinement of EC classification into molecular subgroups has significantly influenced therapeutic strategies, positioning immunotherapy as a central component in the treatment of patients with EC, as reflected in the latest ESGO–ESTRO–ESP clinical practice guidelines [11]. Among these subgroups, dMMR/MSI-H tumors exhibit high immunogenicity due to their elevated TMB and increased neoantigen presentation, which together facilitate robust activation of the immune response. This molecular profile promotes the recruitment of T cells to the tumor microenvironment, resulting in substantial immune cell (IC) infiltration. However, tumor cells frequently overexpress PD-L1, which interacts with PD-1 receptors on T cells, leading to immune evasion through T cell exhaustion [12].

ICIs, including PD-1 inhibitors (e.g., pembrolizumab, nivolumab, dostarlimab) and PD-L1 inhibitors (e.g., atezolizumab, durvalumab), have revolutionized the treatment of dMMR/MSI-H tumors by blocking the PD-1/PD-L1 interaction. This blockade reactivates cytotoxic T cells, restoring their capacity to recognize and eliminate malignant cells [13].

The clinical efficacy of ICIs was first demonstrated in the KEYNOTE-158 trial, which led to the FDA approval of pembrolizumab in 2017 for the treatment of dMMR/MSI-H solid tumors, including EC [14]. Subsequent studies, such as the GARNET trial, confirmed the effectiveness of dostarlimab in patients with dMMR EC, further solidifying the role of ICIs in this subgroup [15].

In contrast, proficient MMR and microsatellite stable (MMRp/MSS) tumors accumulate significantly fewer mutations, resulting in a limited neoantigen landscape and a relatively “cold” immune microenvironment. As a result, the response to ICIs in this subgroup is often limited due to insufficient T-cell priming and activation [16]. This reduced immune responsiveness provides a strong rationale for combination strategies aimed at sensitizing these tumors to immunotherapy. One approach involves combining ICIs with chemotherapeutic agents, which not only induce tumor cell death but also promote the release of tumor neoantigens and inflammatory signals, potentially enhancing antitumor immunity [17].

Another promising strategy includes the use of antiangiogenic agents, which target tumor-induced angiogenesis, a process largely driven by vascular endothelial growth factor (VEGF), that contributes to immunosuppressive conditions within the tumor microenvironment. By inhibiting angiogenesis, these agents can normalize the vasculature and recondition the tumor milieu, making it more permissive to immune infiltration and ICI efficacy [18–21].

A further area of exploration is the synergistic potential between ICIs and DNA-damage response (DDR) targeting agents, such as the poly-ADP-ribose polymerase inhibitors (PARPi). PARPi induce DNA damage and promote cGAS-STING pathway activation, which enhances innate immune responses, increases PD-L1 expression, and supports T cell-mediated cytotoxicity, thereby amplifying the immunogenic effects of ICIs [22].

**Figure 1:**
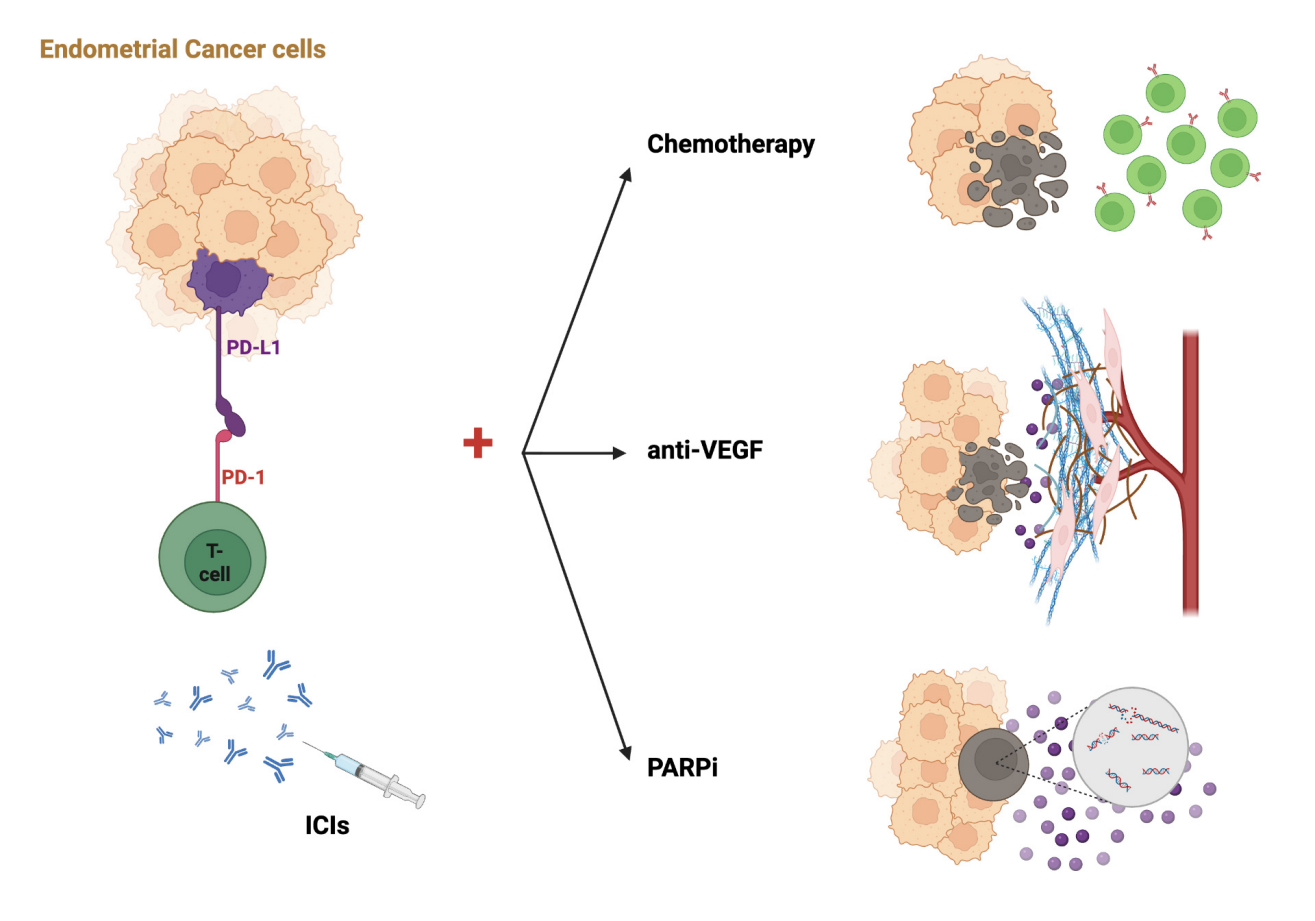
**Mechanisms underlying combinatorial strategies to enhance the efficacy of immune checkpoint inhibitors (ICIs) in endometrial cancer.** Endometrial cancer cells can evade immune surveillance by overexpressing PD-L1, which binds to PD-1 receptors on T cells, leading to T cell exhaustion. ICIs block this interaction, restoring antitumor T cell activity. Chemotherapy promotes immunogenic cell death, facilitating the release of neoantigens and inflammatory mediators. Anti-vascular endothelial growth factor (VEGF) agents counteract tumor-driven angiogenesis and reprogram the tumor microenvironment to promote IC infiltration. Poly-ADP-ribose polymerase inhibitors (PARPi) induce DNA damage and activate the cGAS-STING pathway, enhancing type I interferon signalling, upregulating PD-L1 expression, and promoting T cell-mediated cytotoxicity, thus synergizing with ICIs. Created in BioRender. Colombo, I. (2026) https://BioRender.com/vdt5vfc

Overall, to improve treatment outcomes across these molecular subtypes, especially in the immunologically “cold” MMRp/MSS population, various combination strategies involving ICIs with chemotherapy agents, antiangiogenics, and PARPi are under active investigation, aiming to boost tumor immunogenicity and overcome resistance mechanisms (Figure 1). Emerging data from recent clinical trials have shown promising activity, supporting the potential of these multimodal approaches to expand the benefit of immunotherapy to a broader patient population [23, 24].

## Key clinical trials of immunotherapy in EC

The evolution of immunotherapy in the treatment of patients with EC has been marked by several clinical trials evaluating ICIs in both platinum-pretreated (Table 1) and platinum-naïve a/rEC. The primary goal of these trials has been to determine the efficacy of ICIs in improving outcomes across molecular subgroups, particularly dMMR/MSI-H tumors, which have demonstrated significant responsiveness to immunotherapy, compared to MMRp/MSS tumors, in which benefits remain more limited.

**Table 1:** Study designs and outcomes of key clinical trials of immunotherapy in a/rEC after failure of platinum-based chemotherapy.

**Trial**	**Phase**	**Population**	***N* pts**	**Treatment Arms**	**R**	**Primary Endpoints**	**mFU**	**Outcomes**
KEYNOTE-158 NCT02628067 [14]	II	dMMR a/rEC (cohorts D and K)	90	Pembrolizumab (P) monotherapy	NA	ORR	42.6 m	ORR 48%; DOR NR; PFS 13.1 m; OS NR
GARNET NCT02715284 [25]	I	a/rEC (cohorts A1 dMMR and A2 MMRp)	153 dMMR 161 MMRp	Dostarlimab (Do) monotherapy	NA	ORR, DOR	27.6 m	ORR dMMR 45.5%; MMRp 15.4% DOR dMMR NR; MMRp 19.4 m ORR by CPS ≥ 1: dMMR 54.9%; MMRp 21.7% ORR by MTB: dMMR 47.8%; MMRp 45.5%
KEYNOTE-775 NCT03517449 [23]	III	a/rEC	827	Lenvatinib + Pembrolizumab (L + P) vs. CTX	1:1	PFS, OS in dMMR and ITT	12.2 m (L + P); 10.7 m (CTX)	PFS MMRp: L + P 6.6 m vs. CTX 3.8 m; HR 0.60; *p* < 0.001 PFS ITT: L + P 7.2 m vs. CTX 3.8 m; HR 0.56; *p* < 0.001 OS MMRp: L + P 17.4 vs. CTX 12.0 m; HR 0.68; *p* < 0.001 OS ITT: L + P 18.3 m (15.2–20.5) vs. CTX 11.4 m; HR 0.62; *p* < 0.001

a/rEC: advanced and recurrent endometrial cancer; CPS: combined positive score; CTX: chemotherapy (platinum plus paclitaxel); dMMR: defective mismatch repair; DOR: duration of response; HR: hazard ratio; ITT: intention-to-treat; mFU: median follow-up; MMRp: proficient mismatch repair; MTB: mutational tumor burden; NA: not applicable; NR: not reached; ORR: objective response rate; OS: overall survival; PFS: progression-free survival; R: randomization.

### Immunotherapy in a/rEC after failure of platinum-based chemotherapy

One of the pivotal studies in immunotherapy for a/rEC was the KEYNOTE-158 trial. The trial confirmed that pembrolizumab monotherapy provided durable responses, with an objective response rate (ORR) of 42.6% in dMMR EC patients [14]. This breakthrough paved the way for additional trials evaluating the efficacy of ICIs.

The GARNET trial examined the efficacy of dostarlimab, an anti-PD-1 agent, in patients with dMMR/MSI-H and MMRp/MSS EC. In the dMMR subgroup, the results demonstrated a 45.5% ORR with long-lasting responses, supporting the clinical benefit of immunotherapy in this molecular subgroup. The trial provided a strong basis for dostarlimab’s approval in dMMR a/rEC, reinforcing the role of ICIs as a standard treatment for these tumors. Notably, minimal clinical benefit was observed in patients with MMRp/MSS tumors, with an ORR of only 15.4% and generally less durable responses, further emphasizing the importance of molecular profiling in guiding immunotherapy use [15]. For MMRp/MSS EC, where response rates to ICIs as monotherapy were limited, KEYNOTE-775 evaluated a combination approach using pembrolizumab plus lenvatinib, a multitarget antiangiogenic. The study showed a significant improvement in progression-free survival (median PFS 7.2 vs. 3.8 months, HR 0.56, *p* < 0.001) and overall survival (median OS 18.3 vs. 11.4 months, HR 0.62, *p* < 0.001) compared to chemotherapy, establishing this combination as the preferred second-line treatment for MMRp/MSS a/rEC [23].

### Immunotherapy in first-line a/rEC

Given the promising results in pretreated patients, first-line immunotherapy strategies have been explored in large-scale phase III trials, where ICIs were integrated into platinum-paclitaxel chemotherapy regimens.

The MITO END-3 trial, a phase II randomized, open-label study conducted in 31 Italian Centers, compared carboplatin-paclitaxel with or without avelumab in first-line treatment of a/r EC. In the intention-to-treat (ITT) population, no significant difference in PFS was observed (9.9 vs. 9.6 months; HR 0.78). However, a clinically relevant benefit of chemoimmunotherapy was reported in the dMMR/MSI-H subgroup, with improved 12-month PFS (60% vs. 35%) and 24-month OS rates (76% vs. 55%), and a significant interaction by MMR status for both endpoints (*p* interaction = 0.015 for PFS and 0.029 for OS). The study suggests a benefit from adding avelumab in patients with dMMR/MSI-H tumors. Notably, MMR status was not used as a stratification factor, and the proportion of dMMR patients enrolled (47%) was higher than typically reported in the literature (25–30%) [26, 27].

The RUBY Part 1 trial assessed dostarlimab combined with chemotherapy in first-line a/rEC. Results showed a significant PFS improvement in dMMR/MSI-H patients (HR 0.28), along with a notable benefit in the ITT population (HR 0.64) [28]. Updated data showed a substantial OS advantage, particularly in dMMR/MSI-H tumors (HR 0.30), and a clinically relevant OS benefit also in the MMRp/MSS population (HR 0.79). These results supported the approval of dostarlimab plus chemotherapy in an all-comers population [29, 30]. Consistently, post hoc analyses from RUBY Part 1 showed that dostarlimab plus carboplatin-paclitaxel achieved the longest OS in MMRp/MSS patients, irrespective of subsequent therapies, reinforcing its role as a standard first-line option [30].

Similarly, the NRG-GY018 trial, evaluating pembrolizumab as first-line therapy, confirmed a strong PFS benefit in dMMR/MSI-H patients (HR 0.30), and a more modest yet statistically significant benefit in MMRp/MSS patients (HR 0.54) [31]. Updated OS analyses further demonstrated a survival benefit with the addition of immunotherapy, both in dMMR/MSI-H (HR 0.55) and in the MMRp/MSS subgroup (HR 0.79), reinforcing the role of chemo-immunotherapy as a new standard of care irrespective of MMR status [32]. In addition, updated data submitted to the EMA within the pembrolizumab regulatory dossier reported a PFS HR of approximately 0.74 and an OS HR of 0.80 in MMRp/MSS EC.

Direct comparisons across trials should be interpreted cautiously due to differences in inclusion criteria, histological composition, platinum-free interval definitions, and molecular subgroup distribution.

The RUBY Part 2 trial evaluated dostarlimab plus niraparib as maintenance after first-line chemotherapy in a/rEC. In the overall population, the combination reduced the risk of progression or death by 40% (HR 0.60, 95% CI 0.43–0.82), with a median PFS of 14.5 vs. 8.3 months. A benefit was also seen in the MMRp/MSS subgroup, with a 37% risk reduction (HR 0.63, 95% CI 0.44–0.91) and a 6.0-month gain in median PFS (14.3 vs. 8.3 months) [33]. In contrast, the DUO-E trial included separate treatment arms with durvalumab alone and durvalumab plus olaparib, allowing a more direct assessment of the potential contribution of PARPi to clinical outcomes. Thus, it is not possible to understand the value of the addition of a PARPi to the chemo-immunotherapy combination.

The AtTEnd trial investigated atezolizumab in combination with chemotherapy, demonstrating that dMMR/MSI-H patients achieved a marked reduction in risk of progression (HR 0.36), though benefits in the ITT population were more moderate (HR 0.74). Notably, no significant benefit was observed in the MMRp/MSS subgroup (HR 0.92) [34]. Updated overall survival data did not show a statistically significant OS benefit, confirming the limited impact of this approach beyond the dMMR/MSI-H population [35].

The DUO-E trial, a randomized three-arm study, evaluated chemotherapy alone versus chemotherapy combined with durvalumab, with or without olaparib, followed by maintenance therapy with durvalumab alone or durvalumab plus olaparib. The study met its dual primary endpoints for PFS in the ITT population, demonstrating an HR of 0.71 for the durvalumab arm and 0.55 for the durvalumab plus olaparib arm compared with chemotherapy alone. The greatest benefit of CP plus durvalumab versus CP was observed in the population (HR 0.41). In the MMRp/MSS subgroup, the addition of olaparib maintenance to durvalumab further enhanced PFS outcomes (HR 0.57) [24]. The MMRp/MSS population was highly heterogeneous, with frequent overlap of biomarkers and histological subtypes; notably, 84% of patients in the biomarker-known population were positive for at least one biomarker, and 79% of ctDNA-evaluable patients had detectable ctDNA at baseline. Importantly, the PFS benefit associated with the addition of olaparib maintenance was consistently observed across a range of biomarker-defined and histological subgroups, including patients with detectable ctDNA at baseline, supporting a broader antitumor activity of the chemo-immunotherapy plus PARPi strategy in pMMR/MSS disease [24, 36, 37]. Consequently, the independent contribution of PARP inhibition remains difficult to isolate. Several clinical trials have evaluated the role of ICIs in platinum-naïve a/rEC, with key studies summarized in Table 2.

**Table 2:** Study designs and outcomes of key clinical trials of immunotherapy in 1L a/rEC.

**Trial**	**Phase**	**Population**	***N* pts**	**Treatment Arms**	**R**	**Primary endpoints**	**mFU**	**Outcomes**
DUO-E NCT04269200 [24]	III	1L a/r EC ≥ 12 m after platinum carcinosarcoma included	718	Durvalumab (D), Durvalumab + Olaparib (D+O) vs. Placebo (PBO) + CXT	1:1:1	PFS ITT [D vs. PBO], PFS ITT [D+O vs. PBO]	22 m	PFS ITT: D 10.2 m vs. PBO 9.6 m; HR 0.71; *p* = 0.003; D + O 15.1 m vs. PBO 9.6 m; HR 0.55; *p* < 0.0001^*^ OS ITT: D NR vs. PBO 25.9 m; HR 0.77; *p* = 0.120; D + O NR vs. PBO 25.9 m; HR 0.59; *p* = 0.003 PFS dMMR: D vs. PBO HR 0.42; D + O vs. PBO HR 0.41 PFS MMRp: D vs. PBO HR 0.77; D + O vs. PBO HR 0.57 PFS PD-L1+: D vs. PBO HR 0.63; D + O vs. PBO 0.42
MITO END 3 NCT03503786 [27]	II	1L a/r EC carcinosarcoma excluded	125	Avelumab (Av) vs. PBO + CXT	1:1	PFS ITT	23 m	PFS ITT: Av 9.9 vs. 9.6 m; HR 0.78 12-month PFS dMMR: 60% vs. 35% (*p* = 0.015) 24-month OS dMMR: 76% vs. 55% (*p* = 0.029)
RUBY part 1 NCT04853576 [28]	III	1L a/r EC ≥ 6 m after platinum clear cell, carcinosarcoma, serous, mixed, IIIC2-IVA included	494	Dostarlimab (Do) vs. Placebo (PBO) + CTX	1:1	PFS dMMR-, PFS ITT and OS ITT	24 m	PFS dMMR: Do 61.4% vs. PBO 15.7%; HR 0.28; *p* < 0.001 PFS ITT: Do 36.1% vs. PBO 18.1%; HR 0.64 (0.51–0.80); *p* < 0.001 OS ITT: Do 71.3% vs. PBO 56%; HR 64 (0.46–0.87); *p* = 0.0021 PFS MMRp: Do 28.4% vs. PBO 18.8%; HR 0.76 OS dMMR: Do 83.3% vs. PBO 58.7; HR 0.30 OS MMRp: Do 67.7% vs. PBO 55.1%; HR 0.79
NRG-GY018 NCT03914612 [31, 32]	III	1L a/r EC ≥ 12 m after platinum carcinosarcoma excluded	816	Pembrolizumab (P) vs. Placebo (PBO) + CTX	1:1	PFS dMMR, PFS MMRp	12 m	dMMR PFS: P NR vs. PBO 7.6 m; HR 0.30; *p* < 0.001 MMRp PFS: P 13.1 m vs. PBO 8.7 m; HR 0.54; *p* < 0.001 dMMR OS: HR 0.55 MMRp OS: HR 0.79
RUBY part 2 NCT03981796 [33]	III	1L a/r EC ≥ 6 m after platinum clear cell, carcinosarcoma, serous, mixed, IIIC2-IVA included	291	Dostarlimab (Do) + Niraparib (N) vs. Placebo (PBO) + CTX	2:1	PFS, ITT	NA	PFS ITT: Do + Nira 14.5 vs. 8.3; HR 0.60 PFS dMMR: HR 0.45 PFS MMRp: HR 0.63
AtTEnd NCT03603184 [34]	III	1L a/rEC and ≥ 6 m after platinum carcinosarcoma included	551	Atezolizumab (A) vs. Placebo (PBO) + CXT	2:1	PFS dMMR > PFS ITT > OS ITT	28.3 m	PFS dMMR: A NR vs. PBO 6.9 m; HR 0.36; *p* = 0.0005 PFS ITT: A 10.1 m vs. PBO 8.9 m; HR 0.74; *p* = 0.022 OS ITT: A 38·7 m vs. PBO 30.2 m; HR 0.82; log-rank *p* = 0.048 PFS MMRp: HR 0.92 OS MMRp: HR 1.00
LEAP-001 NCT03884101 [38]	III	1L a/r EC ≥ 6 m after platinum	842	Pembrolizumab (P) + Lenvatinib (L) vs. CTX	1:1	PFS ITT, OS ITT	38.4 m	PFS MMRp: P + L 9.6 vs. CTX 10.2 m; HR 0.99 PFS ITT: P + L 12.5 vs. CTX 10.2 m; HR 0.91 OS MMRp: P + L 30.9 vs. 29.4 m HR 1.02 OS ITT: 37.7 vs. 32.1 m HR 0.93

1L: first line; a/rEC: advanced and recurrent endometrial cancer; CTX: chemotherapy (platinum plus paclitaxel); D: durvalumab; dMMR: defective mismatch repair; HR: hazard ratio; ITT: intention-to-treat; mFU: median follow-up; MMRp: proficient mismatch repair; NA: not applicable; NR: not reached; OS: overall survival; PD-L1: programmed death-ligand 1; PFS: progression-free survival; R: randomization. ^*^ The PFS comparison between Durvalumab alone and placebo was not formally tested for statistical significance according to the predefined hierarchical testing strategy of the DUO-E trial.

The phase III LEAP-001 trial was the first to evaluate a chemoth-free combination, pembrolizumab plus lenvatinib, as first-line treatment for patients with a/rEC, compared to standard carboplatin-paclitaxel. The study did not meet the prespecified statistical criteria for PFS or OS, neither in the MMRp/MSS subgroup nor in the overall population. In the MMRp/MSS population, median PFS was 9.6 months with pembrolizumab-lenvatinib vs. 10.2 months with chemotherapy (HR 0.99), and median OS was 30.9 vs. 29.4 months (HR 1.02). In the ITT population, PFS was 12.5 vs. 10.2 months (HR 0.91), and OS was 37.7 vs. 32.1 months (HR 0.93). The trial did not meet the criteria for superiority in PFS at interim analyses (IA1 or IA2), nor for non-inferiority in OS at final analysis [38]. These findings also underline challenges associated with chemotherapy-free approaches, including patient selection and toxicity management. The results of LEAP-001 further suggest that, despite the established efficacy of ICI plus anti-angiogenic therapy in pretreated MMRp/MSS EC, chemotherapy may represent a critical immunogenic backbone in the treatment-naïve setting, potentially enhancing immune priming and improving responsiveness to immunotherapy.

These studies collectively confirm that ICIs significantly improve PFS and OS in dMMR/MSI-H EC, establishing them as a key component of first-line treatment. In the MMRp/MSS tumors, despite an improvement in patients’ outcomes having been reported, the magnitude of benefit is lower. Combination strategies, such as ICIs with chemotherapy and targeted agents, are being explored to enhance immunogenicity in these less responsive tumors.

## Biomarkers predictive of response to immunotherapy in EC

### MMR/MSI: a key but complex predictive biomarker

**Figure 2:**
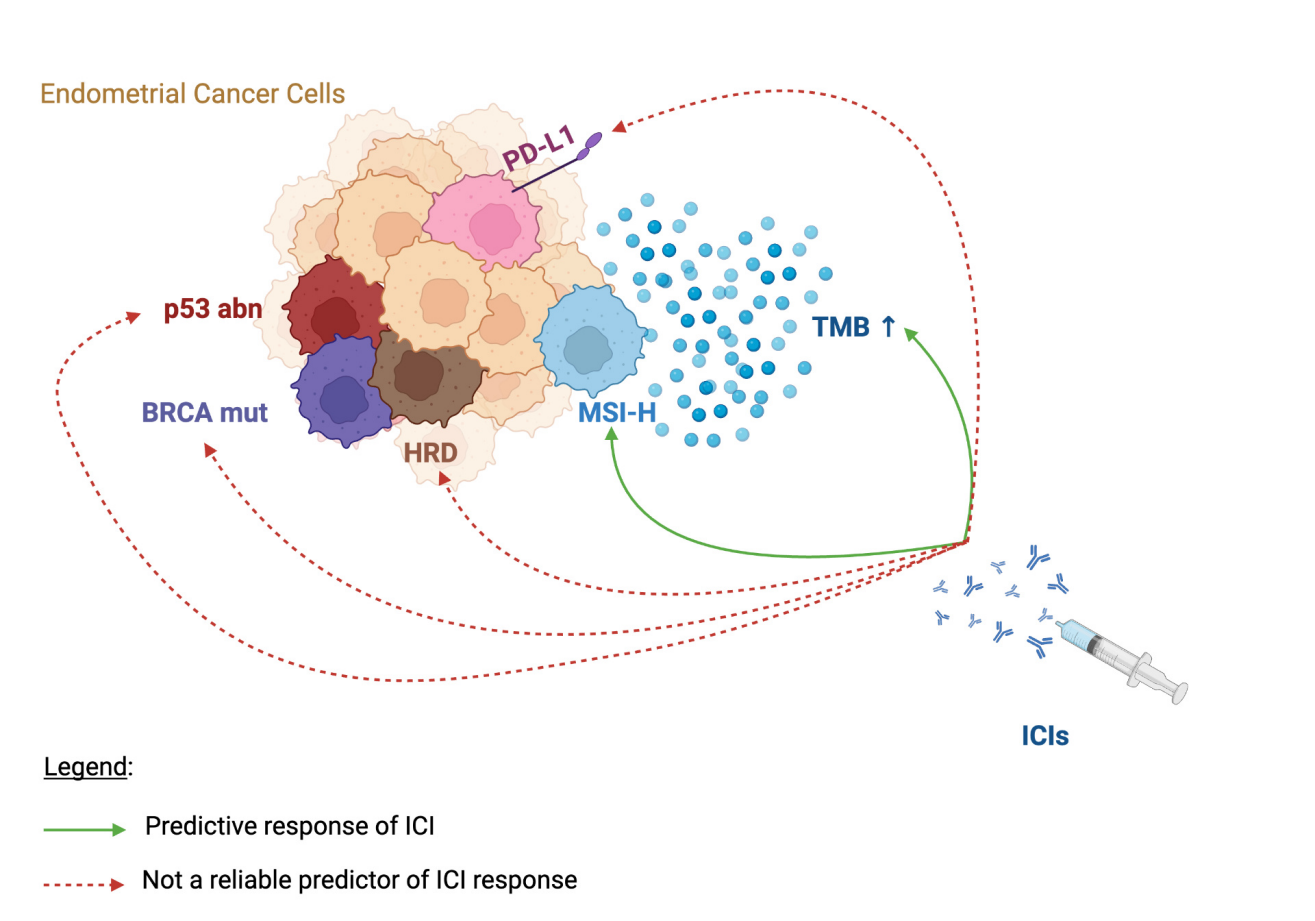
**Biomarkers of response to immune checkpoint inhibitors (ICIs) in endometrial cancer (EC) with or without poly-ADP-ribose polymerase inhibitors (PARPi).** MSI-H EC cells (blue cell) and high tumor mutational burden (TMB, blue points) are predictive biomarkers of ICIs treatment due to their higher neoantigen load. In contrast, MSI-H EC cells (blue cell) and high TMB (blue points) are predictive biomarkers of ICIs due to their higher neoantigen load. In contrast, homologous recombination deficiency (HRD) status, *BRCA* alterations, and possibly TP53 status may represent emerging stratification factors for combination strategies involving ICIs and PARPi, whereas their predictive role for ICIs alone remains unproven. PD-L1 expression currently lacks sufficient evidence as a validated predictive biomarker for ICIs in EC. Arrows represent predictive (green) and Inconsistent/Non-standardized Biomarker (red dashed) of response to ICI monotherapy. Created in BioRender. Colombo, I. (2026) https://BioRender.com/gik2ooa

MMR deficiency is a well-established predictive biomarker for immunotherapy response in EC (Figure 2 — green arrow). The loss of function in MMR proteins (MLH1, PMS2, MSH2, MSH6) leads to MSI-H, resulting in a high TMB and increased neoantigen load, which enhances immune recognition and response to PD-1 inhibitors. This has been confirmed in clinical trials such as GARNET and KEYNOTE-158, supporting the approval of dostarlimab and pembrolizumab [25, 39].

However, emerging evidence indicates that not all dMMR/MSI-H tumors behave identically. The underlying mechanism of MMR deficiency, whether due to germline mutations (e.g., Lynch syndrome), somatic mutations**,** or more commonly, epigenetic *MLH1* promoter hypermethylation (*MLH1*ph), may influence immunotherapy outcomes [40, 41]. Tumors with *MLH1*ph tend to present at more advanced stages, with higher rates of lymphovascular space invasion (LVSI) and fewer tumor-infiltrating lymphocytes (TILs), potentially explaining their reduced sensitivity to PD-1 blockade compared to tumors with genetic MMR alterations [41, 42]. Chow et al. [42] found that among ICI responders, patients with genetic mutations (Mut-dMMR responders) exhibited a strong CD8+ T-cell response, which was further enhanced by PD-1 inhibition. In contrast, responders with epigenetic alterations (epi-dMMR responders) relied more on natural killer (NK) cells, particularly the CD16+ NK cell population. These findings suggest that immune responses to immunotherapy may differ between these groups, yet both can benefit from treatment due to the distinct immune profiles of their tumors.

Several clinical trials have explored whether these biological differences translate into differences in patient outcomes. The NRG-GY018 trial showed a slightly better 12-month PFS in patients with MMR gene mutations (85%) compared to those with *MLH1*ph (75%), although not statistically significant [43]. Likewise, a post-hoc analysis of the GARNET trial found no meaningful difference in ORR across dMMR subgroups, suggesting that dostarlimab is effective regardless of the cause of MMR deficiency [44]. Consistently, exploratory analyses from the RUBY trial did not show clinically relevant differences in efficacy between dMMR tumors with *MLH1* hypermethylation and those with MMR gene mutations, supporting a class effect of immune checkpoint inhibition across dMMR subtypes [45].

Despite these findings, although all dMMR EC tumors generally benefit from immunotherapy, the magnitude of the response varies, and the precise reasons remain unclear [41]. Emerging evidence also suggests that developmental signaling pathways, including Sonic Hedgehog (SHH), may contribute to shaping the tumor immune microenvironment in EC. Recent bioinformatic analyses have associated SHH pathway activation with increased CD8+ T-cell and NK-cell infiltration and improved survival outcomes, supporting its potential role as an emerging biomarker for immune contexture and patient stratification. However, further validation is required before clinical application. Moreover, although several biological mechanisms have been proposed, the determinants of primary resistance to ICIs in dMMR EC remain incompletely understood and are not yet clinically actionable [46]. Further research is needed to determine whether refining dMMR classifications could enhance treatment personalization and optimize outcomes [41].

### TMB and its role in immunotherapy response

The TMB has been investigated as a potential biomarker for response to ICIs, as it reflects the neoantigen load, which enhances immune recognition. However, its predictive value is not consistent across all cancer types. The KEYNOTE-158 trial demonstrated that EC with high TMB had an ORR four times higher than those with low TMB, supporting its role as a predictor of immunotherapy response. This led to the FDA approval of pembrolizumab for TMB-high tumors, though no significant correlation was found between TMB and PD-L1 expression [39]. Higher TMB was associated with increased CD8+ T-cell infiltration, neoantigen load, and improved ORR in the GARNET trial regardless of MSS status (86.5% of MMRd/MSI-H tumors were TMB-high, compared to only 7% of MMRp/MSS tumors), suggesting a possible association with response to ICIs, although its independent predictive value remains uncertain (Figure 2 — green arrow) [25]. The substantial variability in assays, cut-offs, and scoring systems limits reproducibility and currently prevents PD-L1 implementation as a validated predictive biomarker in EC.

In this regards, TMB assessment is challenged by a lack of standardization. Different assays and thresholds, such as the ≥ 10 mutations/megabase cutoff from Foundation Medicine, result in inconsistent outcomes across trials and Centers. Several studies highlight differences in gene coverage, bioinformatics pipelines, and significant variability in defining TMB positivity affecting comparability in clinical trials [47–49]. These discrepancies underscore the need for standardized assays and well-defined thresholds to effectively incorporate TMB as a stratification criterion in clinical trials. This will be critical for clearly defining its role in EC and optimizing its use as an already established predictive biomarker for immunotherapy [50].

### PD-L1 expression: variability in cut-offs, assays, and possible role in EC

The role of PD-L1 and PD-1 as prognostic and predictive biomarkers in EC is still uncertain. Although PD-1 plays a role in suppressing autoimmunity and promoting self-tolerance, variability in assays and scoring systems has made it difficult to define PD-L1 as a reliable predictive biomarker, with expression levels ranging from 5% to 76% across studies [51, 52]. A post-hoc analysis of the GARNET trial used the combined positive score (CPS, cut-off = 1) to evaluate PD-L1 expression in both dMMR/MSI-H and MMRp/MSS tumors. There was no significant difference in response to ICIs between PD-L1-positive and negative groups. However, patients with both high TMB and PD-L1 positivity showed better response rates, suggesting that TMB-high may be a stronger predictive marker than PD-L1 alone [25]. The DUO-E trial used the Tumor Area Positivity (TAP) score to assess PD-L1 status and observed that patients with TAP ≥ 1 had improved PFS in both the immunotherapy alone and immunotherapy + olaparib arms compared with patients with TAP < 1 [24]. The AtTEnd trial showed that patients with PD-L1-positive IC had a better response to atezolizumab + chemotherapy than chemotherapy alone [34].

These trials used different assays and scoring systems (CPS, IC, tumor proportion score [TPS], TAP), highlighting the lack of a standardized approach for PD-L1 assessment [53]. Among them, CPS, IC, and TAP scores appear to be more reliable, as they account for both IC and TPS, thereby reflecting the broader role of the tumor microenvironment and its interaction with the immune system in therapeutic response. From a molecular perspective, dMMR tumors tend to have high PD-L1 expression, supporting their sensitivity to immunotherapy. Conversely, p53-mutated and *POLE*-mutated subgroups show no clear association with PD-L1 positivity [53].

Despite extensive research, PD-L1 remains an inconsistent biomarker due to variability in expression, assay techniques, and cut-off definitions (Figure 2 — red dashed arrow) [52]. Current data suggest that evaluating PD-L1 in both tumor and ICs is more informative than tumor expression alone. These findings reinforce the idea that the tumor microenvironment plays a central role in immunotherapy response, emphasizing the need for a standardized approach for PD-L1 assessment before it can be widely used as a predictive marker.

### Emerging biomarkers of immune response in EC: *TP53*, homologous recombination repair (HRR), homologous recombination deficiency (HRD), and *BRCA*

HRR is a DNA double-strand break repair mechanism involving proteins encoded by *BRCA1/2* (germline and somatic mutations, and promoter methylation), *RAD51*, *RAD51C*, *RAD51D*, *PALB2*, *ATM*, and *ATR* genes [54]. HRD represents genomic instability resulting from HRR pathway dysfunction, but also detectable through specific genomic scar signatures including telomeric allelic imbalance, large-scale transitions, and loss of heterozygosity [54, 55]. In EC, dMMR and HRD present distinct molecular signatures [56], though emerging data reveal biological intersections between the HRR and MMR pathways, with potential synergistic implications [56]. HRR genes such as *ATM* and *ATR*, which regulate the cell cycle, can upregulate PD-L1 expression. Additionally, defective DNA repair mechanisms and cytosolic DNA accumulation increase neoantigen load and activate type I immunity, respectively, supporting the combined application of PARPi and ICIs [57].

*TP53* mutations critically impact tumor biology through immune activation, cell cycle regulation, apoptosis, and DNA repair [22]. Notably, copy-number high, serous-like EC and High Grade Serous Ovarian Cancer exhibit significant molecular similarities, suggesting shared pro-oncogenic pathways like alteration in *TP53*. Moreover, increasing evidence suggested a role of p53 mutations in DNA damage response [58]. Approximately 34% of ECs frequently display alterations in genes such as *ARID1A* and *BRCA*, leading to HRD status [59]. De Jonge et al. [60] showed that HRD was significantly associated with non-endometrioid histology and *TP53*-mutated ECs, with 100% of HRD-positive ECs harboring *TP53* mutations, while only 46% of *TP53*-mutated tumors are HRD-positive, indicating a strong, though not exclusive, association between the two biomarkers. Additionally, HRD status is more frequently observed in MMRp ECs with *TP53* mutations and high-grade histology, further reinforcing the link between *TP53* alterations and HRD [61]. Given the established sensitivity of HRD-positive tumors to platinum-based chemotherapy and DDR-targeted agents in other malignancies, it is plausible that HRD-positive EC, particularly the serous subtype and p53-mutated, may also benefit from these therapies [60]. In the post-hoc subgroup analysis of the RUBY trial part 1, dostarlimab improved overall survival in MMRp ECs with *TP53* mutations [29]. In contrast, the MITO-END trial found no clinical benefit from Avelumab in MSS tumors with *TP53* mutations, though the analysis was limited by sample size [27]. Finally, the DUO-E trial demonstrated that combining chemotherapy plus durvalumab, with or without olaparib, improved clinical outcomes regardless of HRR status. Notably, subgroup analyses suggested a greater benefit from the addition of olaparib in patients with p53-abnormal tumors [24]. These findings suggest that HRD status and *TP53* alterations may represent biologically relevant stratification factors in combination strategies involving ICIs and PARPi, rather than validated predictive biomarkers of response to immunotherapy alone [24].

Despite variability in study designs and clinical endpoints, current evidence supports HRD status and *BRCA* alterations as biologically relevant biomarkers for sensitivity to DDR-targeted therapies rather than validated predictive biomarkers of immunotherapy response (Figure 2 — red dashed arrows). Although exploratory analyses suggest potential interactions between DNA repair defects and immune activation, their predictive value for ICIs remains uncertain and is currently unsupported by robust prospective clinical evidence. Similarly, the role of *TP53* alterations in predicting immunotherapy response remains controversial and requires further validation (Figure 2 — red dashed arrows). These findings underscore the need for cautious interpretation and additional biomarker-driven studies before clinical implementation.

## Conclusions

Current evidence establishes dMMR/MSI-H as the only validated predictive biomarker for immunotherapy response in EC. However, emerging data highlight biological heterogeneity within this group. Tumors with *MLH1*ph, the most frequent cause of dMMR, exhibit reduced immune infiltration, more advanced stage at diagnosis, and increased LVSI, correlating with a less robust response to ICIs compared to tumors harboring germline or somatic MMR mutations [41, 42]. While *MLH1*ph tumors may rely more on NK cell activity, mutational dMMR/MSI-H tumors show enhanced CD8+ T-cell responses, underlining distinct immune landscapes. Nevertheless, trials like GARNET and KEYNOTE-158 have demonstrated significant clinical benefit with PD-1 blockade across the dMMR/MSI-H population [14, 25].

In contrast, the MMRp/MSS subgroup, traditionally less responsive to immunotherapy, contains a subset of “good responders,” particularly those with high TMB and prominent CD8+ T-cell infiltration [47]. In KEYNOTE-158, TMB-high tumors showed a fourfold increase in ORR versus TMB-low, reinforcing TMB’s predictive potential [39]. However, lack of standardization in TMB assessment limits its clinical utility [50].

PD-L1 expression and a pro-inflammatory tumor microenvironment may further enhance ICI efficacy in MMRp tumors, as suggested by KEYNOTE-775 [23]. Yet, its predictive value remains inconsistent—e.g., in GARNET, PD-L1 status did not correlate with response [25]. These discrepancies emphasize the urgent need for composite biomarkers to better stratify MMRp patients, a group that still represents a therapeutic challenge. Future research should prioritize composite biomarker models integrating molecular alterations, immune profiling, and circulating biomarkers, alongside comprehensive characterization of the tumor microenvironment. Longitudinal immune monitoring and multi-omics approaches may further improve prediction of immunotherapy response, particularly in MMRp/MSS EC where validated predictive biomarkers remain lacking.

To overcome resistance mechanisms in MMRp EC, combination strategies involving ICIs with chemotherapy, anti-angiogenic agents, and PARPi have shown promising preliminary activity [31]. The DUO-E trial demonstrated PFS improvement with durvalumab plus olaparib, regardless of HRD or *BRCA* status [24]. Notably, RUBY Part 1 remains the only study to date to demonstrate a significant OS benefit in the overall population**,** not just dMMR patients, supporting the broader efficacy of immunotherapy in a/r EC [28].

Beyond validated biomarkers such as MSI, TMB, and PD-L1, emerging data suggest that *PIK3CA* and *PTEN* mutations, frequent in EC, may influence immune evasion and response to targeted agents and immunotherapy [22]. Additionally, the tumor immune microenvironment plays a critical role in shaping ICI responsiveness. A recent analysis by Grau Bejar et al. [62] revealed that specific immune cell populations, including T cells and myeloid subtypes, correlate with treatment outcomes and may serve as novel immunological biomarkers.

Several ongoing randomized trials are investigating ICIs in early-stage or first-line settings to expand their benefit and explore predictive signatures. DOMENICA (NCT05201547) and KEYNOTE-C93 (NCT05647558) trials are evaluating ICIs versus chemotherapy in first-line a/rEC [63, 64]. RAINBO umbrella program (NCT05255653) and KEYNOTE-B21 (NCT04634877) trials are assessing adjuvant immunotherapy or combination regimens based on molecular risk profiles [65, 66].

Promising future strategies include immune profiling in the neoadjuvant setting or in “window of opportunity” studies, which allow dynamic assessment of tumor-immune interactions before and after brief ICI exposure. This approach may enhance the discovery of early predictive biomarkers [67, 68]. A phase II study presented at SGO 2024 supports the feasibility of this approach in EC, reporting immune activation following short-term preoperative ICI [69].

Additionally, novel approaches employing antibody-drug conjugates (ADCs) against new targets such as HER2, TROP2, and B7-H4 are under investigation and may further expand therapeutic options for patients with refractory or biomarker-negative disease [70–72].

In conclusion, while immunotherapy has revolutionized the management of a/rEC, particularly in dMMR/MSI-H tumors, significant challenges remain in the MMRp/MSS subgroup. Although TMB and PD-L1 are promising biomarkers, their clinical use is limited by variability and lack of standardization. Moving forward, future efforts should prioritize the development of composite predictive models integrating molecular classification, genomic alterations, immune contexture, and circulating biomarkers, together with tumor microenvironment profiling, to enable more accurate patient stratification and personalized immunotherapy approaches.

Future efforts should prioritize the development of composite predictive models integrating molecular classification, genomic alterations, immune contexture, and circulating biomarkers. Emerging approaches such as tumor microenvironment characterization, spatial immune profiling, and longitudinal ctDNA monitoring may improve prediction of immunotherapy response and enable dynamic treatment adaptation. In parallel, ongoing biomarker-driven trials and investigation of novel immune targets and antibody–drug conjugates are expected to refine patient stratification and expand therapeutic opportunities, particularly in the challenging MMRp/MSS EC subgroup. Furthermore, emerging artificial intelligence (AI)- and machine learning (ML)-based approaches may facilitate integration of molecular, histopathological, imaging, and immune profiling data, supporting the development of multimodal predictive models and more precise stratification for immunotherapy in biologically heterogeneous MMRp/MSS EC populations [73, 74].

## Abbreviations

a/rEC: advanced and recurrent endometrial cancer
CPS: combined positive score
DDR: DNA-damage response
dMMR/MSI-H: defective mismatch repair and microsatellite instability-high
ER: estrogen receptor
HRD: homologous recombination deficiency
HRR: homologous recombination repair
IC: immune cell
ICIs: immune checkpoint inhibitors
ITT: intention-to-treat
LVSI: lymphovascular space invasion
MMRp/MSS: proficient mismatch repair and microsatellite stable
NK: natural killer
NSMP: No Specific Molecular Profile
PARPi: poly-ADP-ribose polymerase inhibitors
PR: progesterone receptor
SHH: Sonic Hedgehog
TAP: Tumor Area Positivity
TMB: tumor mutational burden
TPS: tumor proportion score

## Declarations

### Copyright

© The Author(s) 2026.

## References

[1] Siegel RL, Giaquinto AN, Jemal A (2024). Cancer statistics, 2024. CA: Cancer J Clin.

[2] Bray F, Laversanne M, Sung H, Ferlay J, Siegel RL, Soerjomataram I (2024). Global cancer statistics 2022: GLOBOCAN estimates of incidence and mortality worldwide for 36 cancers in 185 countries. CA: Cancer J Clin.

[3] Oaknin A, Bosse TJ, Creutzberg CL, Giornelli G, Harter P, Joly F (2022). Endometrial cancer: ESMO Clinical Practice Guideline for diagnosis, treatment and follow-up. Ann Oncol.

[4] Bokhman JV (1983). Two pathogenetic types of endometrial carcinoma. Gynecol Oncol.

[5] Kandoth C, Schultz N, Cherniack AD, Akbani R, Liu Y, Shen H, Cancer Genome Atlas Research Network (2013). Integrated genomic characterization of endometrial carcinoma. Nature.

[6] Kommoss S, McConechy MK, Kommoss F, Leung S, Bunz A, Magrill J (2018). Final validation of the ProMisE molecular classifier for endometrial carcinoma in a large population-based case series. Ann Oncol.

[7] Vermij L, Smit V, Nout R, Bosse T (2019). Incorporation of molecular characteristics into endometrial cancer management. Histopathology.

[8] Nero C, Trozzi R, Persiani F, Rossi S, Mastrantoni L, Duranti S (2025). *POLE* mutations in endometrial carcinoma: Clinical and genomic landscape from a large prospective single‐center cohort. Cancer.

[9] Chang YW, Kuo HL, Chen TC, Chen J, Lim L, Wang KL (2024). Abnormal p53 expression is associated with poor outcomes in grade I or II, stage I, endometrioid carcinoma: a retrospective single-institute study. J Gynecol Oncol.

[10] Vermij L, Jobsen JJ, León-Castillo A, Brinkhuis M, Roothaan S, Powell ME (2023). Prognostic refinement of NSMP high-risk endometrial cancers using oestrogen receptor immunohistochemistry. Br J Cancer.

[11] Concin N, Matias-Guiu X, Cibula D, Colombo N, Creutzberg CL, Ledermann J (2025). ESGO–ESTRO–ESP guidelines for the management of patients with endometrial carcinoma: update 2025. Lancet Oncol.

[12] Di Dio C, Bogani G, Di Donato V, Cuccu I, Muzii L, Musacchio L (2023). The role of immunotherapy in advanced and recurrent MMR deficient and proficient endometrial carcinoma. Gynecol Oncol.

[13] Nakajima H, Nakatsura T (2020). Towards the era of immune checkpoint inhibitors and personalized cancer immunotherapy. Immunol Med.

[14] O'Malley DM, Bariani GM, Cassier PA, Marabelle A, Hansen AR, De Jesus Acosta A (2022). Pembrolizumab in Patients With Microsatellite Instability—High Advanced Endometrial Cancer: Results From the KEYNOTE-158 Study. J Clin Oncol.

[15] Oaknin A, Gilbert L, Tinker AV, Brown J, Mathews C, Press J (2022). Safety and antitumor activity of dostarlimab in patients with advanced or recurrent DNA mismatch repair deficient/microsatellite instability-high (dMMR/MSI-H) or proficient/stable (MMRp/MSS) endometrial cancer: interim results from GARNET—a phase I, single-arm study. J ImmunoTher Cancer.

[16] Rizzo A (2022). Immune Checkpoint Inhibitors and Mismatch Repair Status in Advanced Endometrial Cancer: Elective Affinities. J Clin Med.

[17] Salas-Benito D, Pérez-Gracia JL, Ponz-Sarvisé M, Rodriguez-Ruiz ME, Martínez-Forero I, Castañón E (2021). Paradigms on Immunotherapy Combinations with Chemotherapy. Cancer Discov.

[18] Emens LA, Middleton G (2015). The Interplay of Immunotherapy and Chemotherapy: Harnessing Potential Synergies. Cancer Immunol Res.

[19] Lee WS, Yang H, Chon HJ, Kim C (2020). Combination of anti-angiogenic therapy and immune checkpoint blockade normalizes vascular-immune crosstalk to potentiate cancer immunity. Exp Mol Med.

[20] Phillipps J, Zhou AY, Butt OH, Ansstas G (2023). PARP inhibition and immunotherapy: a promising duo in fighting cancer. Transl Cancer Res.

[21] Perez-Fidalgo JA, Martinelli E (2023). Lenvatinib plus pembrolizumab a new effective combination of targeted agents. ESMO Open.

[22] Muñoz-Fontela C, Mandinova A, Aaronson SA, Lee SW (2016). Emerging roles of p53 and other tumour-suppressor genes in immune regulation. Nat Rev Immunol.

[23] Makker V, Colombo N, Casado Herráez A, Santin AD, Colomba E, Miller DS (2022). Lenvatinib plus Pembrolizumab for Advanced Endometrial Cancer. N Engl J Med.

[24] Westin SN, Moore K, Chon HS, Lee J-Y, Pepin JT, Sundborg M (2024). Durvalumab Plus Carboplatin/Paclitaxel Followed by Maintenance Durvalumab With or Without Olaparib as First-Line Treatment for Advanced Endometrial Cancer: The Phase III DUO-E Trial. J Clin Oncol.

[25] Oaknin A, Pothuri B, Gilbert L, Sabatier R, Brown J, Ghamande S (2023). Safety, Efficacy, and Biomarker Analyses of Dostarlimab in Patients with Endometrial Cancer: Interim Results of the Phase I GARNET Study. Clin Cancer Res.

[26] Talhouk A, McConechy MK, Leung S, Yang W, Lum A, Senz J (2017). Confirmation of ProMisE: A simple, genomics-based clinical classifier for endometrial cancer. Cancer.

[27] Pignata S, Scambia G, Schettino C, Arenare L, Pisano C, Lombardi D, MITO investigators (2023). Carboplatin and paclitaxel plus avelumab compared with carboplatin and paclitaxel in advanced or recurrent endometrial cancer (MITO END-3): a multicentre, open-label, randomised, controlled, phase 2 trial. Lancet Oncol.

[28] Mirza MR, Chase DM, Slomovitz BM, dePont Christensen R, Novák Z, Black D (2023). Dostarlimab for Primary Advanced or Recurrent Endometrial Cancer. N Engl J Med.

[29] Powell M, Auranen A, Willmott L, Gilbert L, Black D, Cibula D (2024). 37MO Dostarlimab plus chemotherapy in primary advanced or recurrent endometrial cancer (pA/rEC) in the RUBY trial: Overall survival (OS) by MMR status and molecular subgroups. ESMO Open.

[30] Powell M, Roed H, Gilbert L, Zub O, McCourt C, Fleming E (2025). 1113P Post hoc survival outcomes based on initial and subsequent treatment in patients (pts) with mismatch repair proficient/microsatellite stable (MMRp/MSS) primary advanced or recurrent endometrial cancer (pA/R EC) in the ENGOT-EN6-NSGO/GOG-3031/RUBY trial. Ann Oncol.

[31] Eskander RN, Sill MW, Beffa L, Moore RG, Hope JM, Musa FB (2023). Pembrolizumab plus Chemotherapy in Advanced Endometrial Cancer. N Engl J Med.

[32] Eskander RN, Sill MW, Beffa L, Moore RG, Hope JM, Musa FB (2025). Pembrolizumab plus chemotherapy in advanced or recurrent endometrial cancer: overall survival and exploratory analyses of the NRG GY018 phase 3 randomized trial. Nat Med.

[33] Mirza M, Ghamande S, Hanker L, Black D, Raaschou-Jensen N, Gilbert L (2024). 38MO Progression-free survival (PFS) in primary advanced or recurrent endometrial cancer (pA/rEC) in the overall and mismatch repair proficient (MMR/MSS) populations and in histological and molecular subgroups: Results from part 2 of the RUBY trial. ESMO Open.

[34] Colombo N, Biagioli E, Harano K, Galli F, Hudson E, Antill Y, AtTEnd study group (2024). Atezolizumab and chemotherapy for advanced or recurrent endometrial cancer (AtTEnd): a randomised, double-blind, placebo-controlled, phase 3 trial. Lancet Oncol.

[35] Ginesta MB, Biagioli E, Harano K, Galli F, Hudson E, Antill Y (2025). LBA39 Final overall survival (OS) results from the randomized double-blind phase III AtTEnd/ENGOT-EN7 trial evaluating atezolizumab in combination with paclitaxel and carboplatin in women with advanced/recurrent endometrial cancer. Ann Oncol.

[36] Westin SN, Moore KN, Guy M, Jordan S, McHale M, Miller E (2025). Durvalumab plus carboplatin/paclitaxel followed by durvalumab with or without olaparib as first-line treatment for endometrial cancer: Longitudinal changes in circulating tumor DNA. J Clin Oncol.

[37] Moore K, Westin S, Chon HS, Pepin JT, Salinas E, Starks D (2025). Durvalumab plus carboplatin/paclitaxel followed by durvalumab with/without olaparib in endometrial cancer: Biomarkers, histological heterogeneity, baseline circulating tumor DNA and efficacy in the DUO-E mismatch repair proficient subpopulation. Gynecol Oncol.

[38] Marth C, Moore RG, Bidziński M, Pignata S, Ayhan A, Rubio MJ, ENGOT-en9/LEAP-001 Investigators (2025). First-Line Lenvatinib Plus Pembrolizumab Versus Chemotherapy for Advanced Endometrial Cancer: A Randomized, Open-Label, Phase III Trial. J Clin Oncol.

[39] Marabelle A, Fakih M, Lopez J, Shah M, Shapira-Frommer R, Nakagawa K (2020). Association of tumour mutational burden with outcomes in patients with advanced solid tumours treated with pembrolizumab: prospective biomarker analysis of the multicohort, open-label, phase 2 KEYNOTE-158 study. Lancet Oncol.

[40] Carvalho FM, Carvalho JP (2024). Unraveling the Heterogeneity of Deficiency of Mismatch Repair Proteins in Endometrial Cancer: Predictive Biomarkers and Assessment Challenges. Cancers.

[41] Manning-Geist BL, Liu YL, Devereaux KA, Paula ADC, Zhou QC, Ma W (2022). Microsatellite Instability—High Endometrial Cancers with *MLH1* Promoter Hypermethylation Have Distinct Molecular and Clinical Profiles. Clin Cancer Res.

[42] Chow RD, Michaels T, Bellone S, Hartwich TMP, Bonazzoli E, Iwasaki A (2022). Distinct Mechanisms of Mismatch-Repair Deficiency Delineate Two Modes of Response to Anti-PD-1 Immunotherapy in Endometrial Carcinoma. Cancer Discov.

[43] Eskander R, Sill M, Miller A, Beffa L, Moore R, Hope J (2023). LBA43 Updated response data and analysis of progression free survival by mechanism of mismatch repair loss in endometrial cancer (EC) patients (pts) treated with pembrolizumab plus carboplatin/paclitaxel (CP) as compared to CP plus placebo (PBO) in the NRG GY018 trial. Ann Oncol.

[44] André T, Banerjee S, Berton D, Ellard SL, Jimenez B, Samouëlian V (2022). Abstract 5135: Antitumor activity of dostarlimab by PD-L1 and tumor mutation burden (TMB) in patients (pts) with mismatch repair deficient and proficient (dMMR and MMRp) tumors in the GARNET trial. Cancer Res.

[45] Mirza M, Willmott L, Hietanen S, Gilbert L, Myers T, Balázs B (2025). Updated duration of response for patients receiving dostarlimab plus carboplatin-paclitaxel treatment compared with patients receiving placebo plus carboplatin-paclitaxel in the ENGOT-EN6-NSGO/GOG-3031/RUBY trial. Gynecol Oncol.

[46] Snijesh VP, Krishnamurthy S, Bhardwaj V, Punya KM, Niranjana Murthy AS, Almutadares M (2024). SHH Signaling as a Key Player in Endometrial Cancer: Unveiling the Correlation with Good Prognosis, Low Proliferation, and Anti-Tumor Immune Milieu. Int J Mol Sci.

[47] McGrail DJ, Pilié PG, Rashid NU, Voorwerk L, Slagter M, Kok M (2021). High tumor mutation burden fails to predict immune checkpoint blockade response across all cancer types. Ann Oncol.

[48] Ramos-Paradas J, Hernández-Prieto S, Lora D, Sanchez E, Rosado A, Caniego-Casas T (2021). Tumor mutational burden assessment in non-small-cell lung cancer samples: results from the TMB^2^ harmonization project comparing three NGS panels. J ImmunoTher Cancer.

[49] Fenizia F, Wolstenholme N, Fairley JA, Rouleau E, Cheetham MH, Horan MP (2021). Tumor mutation burden testing: a survey of the International Quality Network for Pathology (IQN Path). Virchows Arch.

[50] Esposito Abate R, Cheetham MH, Fairley JA, Pasquale R, Sacco A, Nicola W (2022). External quality assessment (EQA) for tumor mutational burden: results of an international IQN path feasibility pilot scheme. Virchows Arch.

[51] Nagel J, Paschoalini RB, Barreto PSD, Credidio CH, Paulino E, Del Pilar Estevez-Diz M (2024). Predictive biomarkers in endometrial carcinomas: a review of their relevance in daily anatomic pathology. Surg Exp Pathol.

[52] Mamat Yusof MN, Chew KT, Kampan NC, Shafiee MN (2023). Expression of PD-1 and PD-L1 in Endometrial Cancer: Molecular and Clinical Significance. Int J Mol Sci.

[53] Proppe L, Jagomast T, Beume S, Köster F, Bräutigam K, Rody A (2025). Prognostic and clinical heterogeneity of PD1 and PD-L1- immunohistochemical scores in endometrial cancers. Arch Gynecol Obstet.

[54] Miller RE, Leary A, Scott CL, Serra V, Lord CJ, Bowtell D (2020). ESMO recommendations on predictive biomarker testing for homologous recombination deficiency and PARP inhibitor benefit in ovarian cancer. Ann Oncol.

[55] Takaya H, Nakai H, Takamatsu S, Mandai M, Matsumura N (2020). Homologous recombination deficiency status-based classification of high-grade serous ovarian carcinoma. Sci Rep.

[56] Incorvaia L, Bazan Russo TD, Gristina V, Perez A, Brando C, Mujacic C (2024). The intersection of homologous recombination (HR) and mismatch repair (MMR) pathways in DNA repair-defective tumors. npj Precis Oncol.

[57] Shi Z, Chen B, Han X, Gu W, Liang S, Wu L (2023). Genomic and molecular landscape of homologous recombination deficiency across multiple cancer types. Sci Rep.

[58] Musacchio L, Caruso G, Pisano C, Cecere SC, Di Napoli M, Attademo L (2020). PARP Inhibitors in Endometrial Cancer: Current Status and Perspectives. Cancer Manag Res.

[59] Heeke AL, Pishvaian MJ, Lynce F, Xiu J, Brody JR, Chen WJ (2018). Prevalence of Homologous Recombination–Related Gene Mutations Across Multiple Cancer Types. JCO Precis Oncol.

[60] de Jonge MM, Auguste A, van Wijk LM, Schouten PC, Meijers M, Ter Haar NT (2019). Frequent Homologous Recombination Deficiency in High-grade Endometrial Carcinomas. Clin Cancer Res.

[61] Marquard AM, Eklund AC, Joshi T, Krzystanek M, Favero F, Wang ZC (2015). Pan-cancer analysis of genomic scar signatures associated with homologous recombination deficiency suggests novel indications for existing cancer drugs. Biomark Res.

[62] Grau Bejar JF, Yaniz Galende E, Zeng Q, Genestie C, Rouleau E, de Bruyn M (2024). Immune predictors of response to immune checkpoint inhibitors in mismatch repair-deficient endometrial cancer. J ImmunoTher Cancer.

[63] Joly F, Ray-Coquard IL, Rubio MJ, Paoletti X, Davis AJ, Hudson E (2023). Randomized phase III trial in MMR deficient (MMRd) endometrial cancer (EC) patients comparing chemotherapy (CT) alone versus dostarlimab in first line advanced/metastatic setting: DOMENICA study (GINECO-EN105b/ENGOT-en13 study). J Clin Oncol.

[64] Slomovitz BM, Cibula D, Simsek T, Mirza MR, Maćkowiak-Matejczk B, Hudson E (2022). KEYNOTE-C93/GOG-3064/ENGOT-en15: A phase 3, randomized, open-label study of first-line pembrolizumab versus platinum-doublet chemotherapy in mismatch repair deficient advanced or recurrent endometrial carcinoma. J Clin Oncol.

[65] (2023). RAINBO Research Consortium. Refining adjuvant treatment in endometrial cancer based on molecular features: the RAINBO clinical trial program. Int J Gynecol Cancer.

[66] Van Gorp T, Cibula D, Lv W, Backes F, Ortaç F, Hasegawa K (2024). ENGOT-en11/GOG-3053/KEYNOTE-B21: a randomised, double-blind, phase III study of pembrolizumab or placebo plus adjuvant chemotherapy with or without radiotherapy in patients with newly diagnosed, high-risk endometrial cancer. Ann Oncol.

[67] Eerkens AL, Brummel K, Vledder A, Paijens ST, Requesens M, Loiero D (2024). Neoadjuvant immune checkpoint blockade in women with mismatch repair deficient endometrial cancer: a phase I study. Nat Commun.

[68] Topalian SL, Forde PM, Emens LA, Yarchoan M, Smith KN, Pardoll DM (2023). Neoadjuvant immune checkpoint blockade: A window of opportunity to advance cancer immunotherapy. Cancer Cell.

[69] Lee YJ, Lee YY, Park JY, Cho HW, Lim MC, Kim MK (2025). A phase II study of induction PD-1 blockade (nivolumab) in patients with surgically completely resectable mismatch repair deficient endometrial cancer (NIVEC). J Gynecol Oncol.

[70] Mauricio D, Bellone S, Mutlu L, McNamara B, Manavella DD, Demirkiran C (2023). Trastuzumab deruxtecan (DS-8201a), a HER2-targeting antibody-drug conjugate with topoisomerase I inhibitor payload, shows antitumor activity in uterine and ovarian carcinosarcoma with HER2/neu expression. Gynecol Oncol.

[71] Santin AD, Corr BR, Spira A, Willmott L, Butrynski J, Tse KY (2024). Efficacy and Safety of Sacituzumab Govitecan in Patients With Advanced Solid Tumors (TROPiCS-03): Analysis in Patients With Advanced Endometrial Cancer. J Clin Oncol.

[72] Zhang Z, Jiang C, Liu Z, Yang M, Tang X, Wang Y (2020). B7-H3-Targeted CAR-T Cells Exhibit Potent Antitumor Effects on Hematologic and Solid Tumors. Mol Ther Oncolytics.

[73] Bhardwaj V, Sharma A, Parambath SV, Gul I, Zhang X, Lobie PE (2022). Machine Learning for Endometrial Cancer Prediction and Prognostication. Front Oncol.

[74] Wolde T, Bhardwaj V, Pandey V (2025). Current Bioinformatics Tools in Precision Oncology. MedComm.

